# Typhoid Spine

**Published:** 1919-01

**Authors:** 


**Typhoid Spine.** Sir WILLIAM OSLER. *Bulletin of the Canadian Medical Corps.* September, 1918.

The author records a case of paralysis following an attack of typhoid fever, in which the patient, who had been unable to move from bed for nearly two years, walked after 10 minutes' treatment; and a series of cases with similar symptoms : stiffness and pain in the back, excessive nervousness, hyperesthetic girdle, weak legs and marked basal motor changes. In all these cases, recovery was too rapid for a spondylitis. The author concluded to purely functional disturbance analogous to "railway spine" or "hysterical spine", and his opinion was confirmed by the fact that, in the cases observed, typhoid spine never presented any swelling and never went on to suppuration.

In another series of cases, bone changes were noted in the spine, and in one there was, in addition to the previously described symptoms, a well marked, painful swelling just above the right sacro-iliac articulation. Cases of typhoid spine may, therefore, be divided into the following categories :

(1) Cases in which hysterical features predominate,

(2) Cases with periostitis or perispondylitis with fever, pain, rigidity and nerve-root involvement.

(3) Cases with definite objective changes in the spine.

The patient, whose recovery is complete after 10 minutes of treatment, obviously belongs to class 1.

There has been, in some cases, evidence of actual disease of the spine, which affected two forms : kyphosis and a swelling of the soft parts on either side of the spine.

